# The Effects and Possible Mechanisms of Puerarin to Treat Endometriosis Model Rats

**DOI:** 10.1155/2015/269138

**Published:** 2015-03-01

**Authors:** Jin Yu, Li Zhao, Danying Zhang, Dongxia Zhai, Wei Shen, Lingling Bai, Yiqun Liu, Zailong Cai, Ji Li, Chaoqin Yu

**Affiliations:** ^1^Shanghai University of Traditional Chinese Medicine, Shanghai 201203, China; ^2^Department of Gynecology of Traditional Chinese Medicine, Changhai Hospital, Second Military Medical University, Shanghai 200433, China; ^3^Department of Biochemistry and Molecular Biology, Second Military Medical University, Shanghai 200433, China; ^4^Department of Gynecology of Traditional Chinese Medicine, Longhua Hospital, Shanghai University of Traditional Chinese Medicine, Shanghai 200032, China

## Abstract

*Objective*. To explore the effects of puerarin to treat endometriosis (EMT) model rats and the possible regulatory mechanisms. *Methods.* EMT model rats were surgically induced by autotransplantion of endometrial tissues. The appropriate dosage of puerarin to treat EMT model rats was determined by observing the pathologic morphology of ectopic endometrial tissues and by detecting the levels of estradiol (E2) and prostaglandin E2 (PGE2) of both serum and ectopic endometrial tissues. The related genes and proteins of ectopic endometrial tissues were analyzed by Real-time PCR and immunohistochemistry (IHC) to explore the possible mechanisms. *Results*. Puerarin could reduce the levels of E2 and PGE2 and prevent the growth of ectopic endometrium tissues by inhibiting the expression of aromatase cytochrome P450 (p450arom) and cyclooxygenase-2 (cox-2); puerarin could adjust the anabolism of E2 by upregulating the expression of 17*β*-hydroxysteroid-2 (17*β*-hsd-2) and downregulating the expression of 17*β*-hydroxysteroid-1 (17*β*-hsd-1) of the ectopic endometrium tissues; puerarin could increase the expression of ER*β* and improve the inflammatory microenvironment of EMT model rats. *Conclusions.* Our data suggest that puerarin has a therapeutic effect on EMT model rats and could be a potential therapeutic agent for the treatment of EMT in clinic.

## 1. Introduction

Endometriosis (EMT) causes chronic pelvic pain and infertility [1], affects 5%–10% of women in their reproductive ages [2, 3], and is an estrogen-dependent benign disease characterized by extrauterine implantation and ectopic growth of endometrium [4]. The pathogenesis of EMT is still unclear and one of the most popular theories is “ectopic endometrium implantation” proposed by Sampson in 1921. Current treatments, including surgery and hormonal therapy, are often insufficient and imperfect for high rate of relapse and various side effects such as hepatic injury and osteoporosis [5–7]. Therefore, it is necessary to explore novel therapeutic strategies and drugs for reversing the clinical symptoms of EMT patients and improving their quality of life.

In our early clinical practice to treat endometriosis (EMT), a better therapeutic effect was achieved if the formula of traditional Chinese medicine included* Radix puerariae *[8, 9]. Puerarin, extracted from* Radix puerariae*, is widely known as a natural conditioner of selective estrogen receptors (ERs) [10] and has an antiestrogenic effect for its weak estrogenic action by binding to ERs as we have studied in vitro before. This study was to investigate the effects of puerarin to treat the EMT model rats and the possible regulatory mechanisms in vivo, to provide scientific basis for clinical treatment of EMT patients.

## 2. Materials and Methods 

### 2.1. Animals

SPF grade female Sprague-Dawley (SD) rats (body weight 160–180 g; *n* = 140) were purchased from Shanghai Sippr-bk Laboratory Animals co., Ltd. (license: SCXK (HU) 2008-0016) and raised in Fudan university animal center, 25°C constant temperature (Humidity 50%), 12 h light: 12 h dark cyclical alternates, without feeding soybean and alfalfa products. All procedures described here were reviewed and approved by the Ethical Committee of Fudan Medical University.

### 2.2. Drugs

Puerarin (purity ≥ 98%) was purchased from Sigma (batch number: P5555); raloxifene hydrochloride (RLX, purity 98%) was purchased from Sigma (batch number: 1598201); sodium carboxymethyl cellulose (CMC, 800 cps) was purchased from J&K (batch number: 241297).

### 2.3. Rat Model

At the first 10 days, the estrous cyclicality of all rats was checked with histological screening of vaginal exfoliated cells [11] and the rats with irregular estrous cyclicality were eliminated, while the normal ones were prepared for modeling.

The EMT rat model, as Vernon and Wilson reported [12], was induced by autotransplantation of endometrial tissues. The surgery was taken in aseptic conditions and the right part of the uterus tissue about 1 cm long was cut out with surgical scissors; 4 pieces of the endometrium were stripped carefully and stitched to the following parts, respectively, with nonabsorbable suture (USP8/0-2 number, ZENMA): the root of mesentery (a piece of endometrium (3 mm × 3 mm)), the left side of the ovary (a piece of endometrium (3 mm × 3 mm)), and abdominal wall on both sides (a piece of endometrium (5 mm × 5 mm) on the right side and a piece of endometrium (3 mm × 3 mm) on the left side). After surgery, all rats were treated with cephalosporin (0.1 g/per rat) by intraperitoneal injection for 3 days.

### 2.4. Drug Intervention

The successful EMT model rats verified by exploratory laparotomy were randomly divided into five groups: control group (CMC), low-dose group (L-SI), middle-dose group (M-SI), and high-dose group (H-SI), which intraperitoneally received 0.1% CMC (0.01 mL/body weight (g)/day), 5, 20, and 80 mg of puerarin/body weight (kg)/day, respectively, and positive control group (RLX) which orally received RLX (dose: 10 mg/body weight (kg)/day). Both of puerarin and PLX were dissolved in CMC prorata and all rats of the five groups were respectively administered for 4 weeks between 9:00 and 10:00 a.m. every day.

### 2.5. RIA Analysis

Biochemical assessments of rat serum were detected by RIA analysis. The abdominal aortic blood (about 4 mL) was obtained from the experimental rats anaesthetized by 1% pentobarbital sodium (6 mL/kg body mass). In addition, ectopic endometrium tissues were stripped from the implant sites, homogenated with the refiner, and stored at −80°C refrigerator. At last, the levels of E2 and PGE2 of the serum and the homogenate of ectopic endometrial tissues were detected by RIA kits.

### 2.6. Light Microscopic Analysis

Both eutopic endometrium and ectopic endometrium of rats were surgically detached and the wet weight values were obtained from electronic scales. Parts of the tissues were immediately stored in liquid nitrogen and the others were quickly fixed in 10% formalin for 24 h and then dehydrated in increasing concentrations of ethanol, followed by immersion in xylene and embedding in paraffin wax. The endometrial tissue sections paraffin-embedded (4 *μ*m) were stained with hematoxylin-eosin and analyzed under a conventional light microscope. In addition, 3 fields of each endometrial section were selected randomly and the pathological morphology of endometrial tissues was observed. Two investigators, blinded to the sections' origin, independently analyzed the sections under a conventional microscope and took the available pictures.

### 2.7. Real-Time PCR Analysis

Six ectopic endometrial tissues from each group preserved in liquid nitrogen were randomly selected for Real-time PCR analysis. Total RNA was isolated from ovarian tissues using Trizol reagent (Invitrogen, USA) and following the manufacturer's instructions. Total RNA (3 *μ*g) was reversally transcribed with Reverse Transcription Kit (Tatar, USA) as described by the manufacturer. The resulting cDNA was diluted 10-fold in sterile water and aliquots were subjected to Real-time PCR. PCR primer pairs for the analysis were designed and synthetized by Invitrogen Biological Technology Co., Ltd., Shanghai, China (Table 1). Finally, the levels of P450arom, COX-2, 17*β*-hsd-1, and 17*β*-hsd-2 mRNA were, respectively, analyzed by quantitative PCR instrument (Robo Cycler, Biosystems). The relative expression of each target gene compared to *β*-actin was calculated using the 2^ΔΔCt^ method.

### 2.8. IHC Analysis

Immunohistochemistry for P450arom, COX-2, ER-*α*, and ER-*β* of both eutopic endometrium and ectopic endometrium tissues was performed using a two-step EnVision/HRP technique (Dako Cytomation, Denmark) according to the manufacturer's instruction as Hur et al reported [13]. Intensity and extent of staining was scored and five random fields were observed under a light microscope. The expression of proteins was quantitatively evaluated using an Olympus BH2 microscope with computer-aided images analysis system (Qiu Wei Inc, Shanghai, China). The digital images were archived by a digital camera (Nikon 4500, Tokyo, Japan). The positive area and optical density (OD) of positive cells were determined by measuring three randomly selected microscopic fields (25∗10) for each slide. The IHC index was defined as average integral optical density (AIOD) (AIOD = positive area ∗ OD/total area) [14]. The results were scored by two examiners who were blinded to data and the antibodies used in this research are listed in Table 2.

### 2.9. Statistical Analysis

Statistical evaluations were analyzed using the Statistical Package for the Social Sciences (SPSS version 17.0). Values are expressed as means ± SD. One-factor ANOVA and least significant difference *t*-test (LSD *t*-test) statistical analysis methods were used to analyze the differences among groups. *P* < 0.05 was considered to be statistically significant.

## 3. Results

### 3.1. Estrous Cyclicity

**Figure 1:**
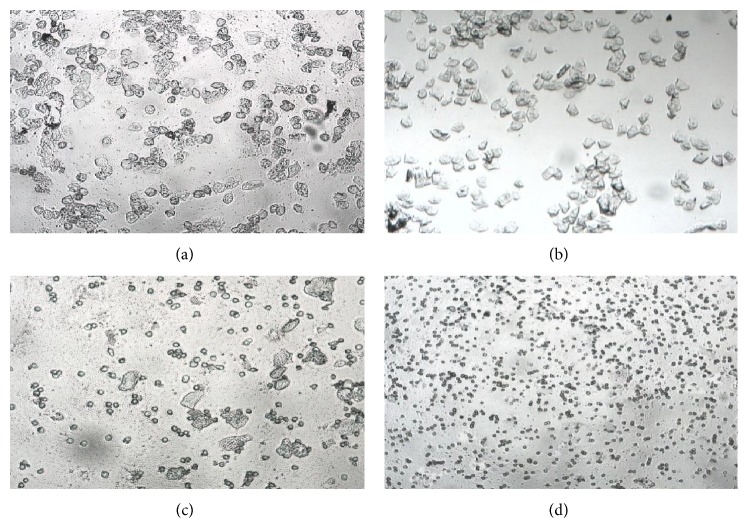
The vaginal exfoliated cells of rats observed by microscopic analysis. (a) Preoestrus: most cells are nucleated epithelial cells. (b) Estrus: large numbers of keratin epithelial cells. (c) Postestrus: several kinds of cells including nucleated epithelial cells, keratin epithelial cells, and leukocyte. (d) Anestrus: large numbers of leukocyte and a small amount of mucus.

According to the classification of vaginal exfoliated cells and the predominant cell type in vaginal smears obtained daily (10 consecutive days) and verified by microscopic analysis, the rats with regular estrous cyclicity were selected for modeling. As a result, 132 rats were up to standard and their estrous cyclicity was showed in Figure 1.

### 3.2. Model Evaluation

**Figure 2:**
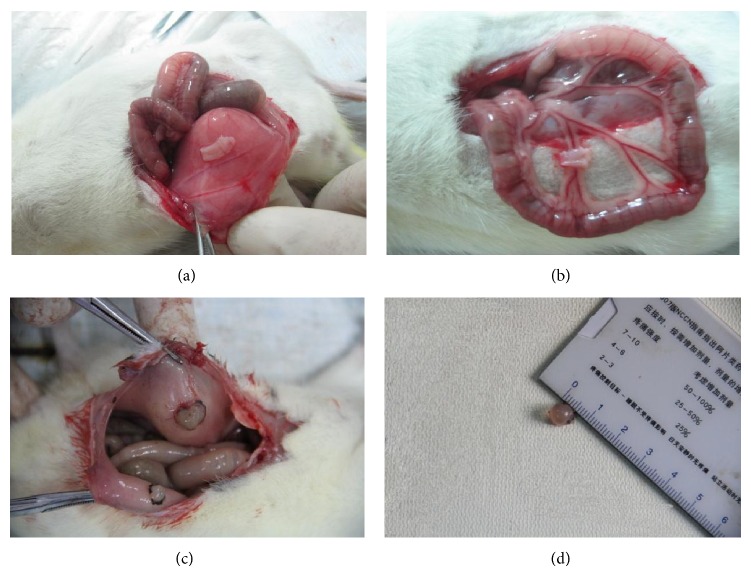
The growth of transplanted endometrial tissues was observed. (a) The endometrium was autotransplanted on the right side of the abdominal wall. (b) The endometrium was autotransplanted on the root of mesentery. Both (a) and (b) showed that the ectopic endometrium tissues were covered by cambium and blood vessels. (c) Endometriosis cysts. (d) The diameter size of endometriosis cysts. Both (c) and (d) showed that the lesions could be nodular and cystic and the cyst was filled with fluid.

**Figure 3:**
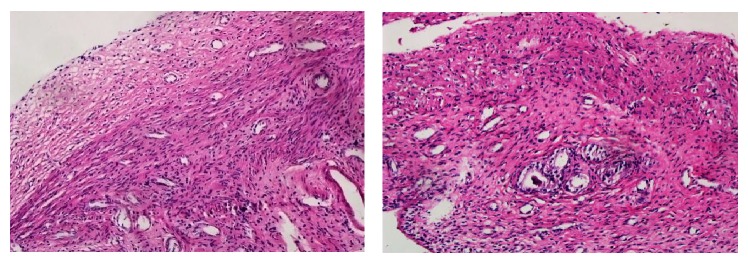
Pathological morphology of the ectopic endometrium tissues before drug intervention analyzed by light microscopic analysis. HE staining showed the growth of epithelial cells, glands, and stroma of autotransplantation endometrial tissues and prompted that the ectopic endometrium has the secretory activity.

132 rats who had normal estrous cyclicity were surgically induced by autotransplantion, however, there were 9 rats died from anesthetization and 24 rats died from infection. 21 days later, 99 rats were verified by exploratory laparotomy. The sites of autotransplantation were surgically exposed and the growth of transplanted endometrial tissues was observed (Figure 2). As a result, 74 rats were successfully induced and another 25 rats were excluded for preoperative anesthesia death, postoperative infection, and other reasons. Thus, the success rate of EMT rat model induced by autotransplantation was 56.06% in our experiment (Table 3). In addition, the ectopic endometrium tissues of 3 rats, randomly, were selected for the pathological morphological observation before drug intervention (Figure 3).

### 3.3. The Weight of Ectopic Endometrium

**Figure 4:**
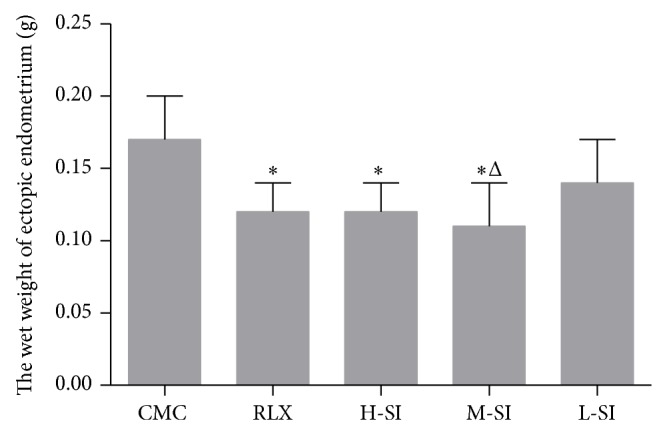
The wet weight value of ectopic endometrium tissues surgically detached from the right side of abdominal wall of every group of rats. As is shown above, compared with CMC group (0.17 ± 0.03, *n* = 6), there were obvious differences in RLX group (0.12 ± 0.02, *n* = 8), H-SI group (0.12 ± 0.02, *n* = 7), and M-SI group (0.11 ± 0.03, *n* = 8), versus CMC group: ^*^ *P* < 0.05, versus L-SI group: ^Δ^ *P* < 0.05.

After drug intervention for 28 days, the ectopic endometrium tissues surgically detached from the right side of abdominal wall of every group rats were weighed and the average wet weight values were compared among groups (Figure 4).

### 3.4. Biochemical Assessments

**Figure 5:**
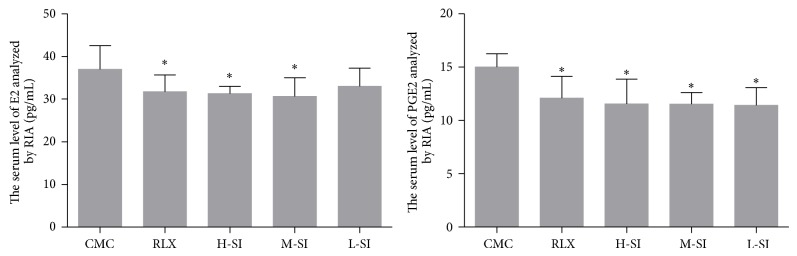
The serum levels of E2 and PGE2 analyzed with RIA. As is shown above, compared with CMC group (37.02 ± 5.49, *n* = 6), the serum levels of E2 in RLX group (31.73 ± 3.95, *n* = 8), H-SI group (31.33 ± 1.63, *n* = 7), and M-SI group (30.66 ± 4.38, *n* = 8) were significantly different, while in L-SI group (32.97 ± 4.28) the difference was not statistically obvious. Besides, the serum levels of PGE2 in RLX group (12.11 ± 2.04, *n* = 8), H-SI group (11.56 ± 2.31, *n* = 7), M-SI group (11.52 ± 1.10, *n* = 8), and L-SI group (11.42 ± 1.65) were statistically different from CMC group (15.03 ± 1.23, *n* = 6), versus CMC group: ^*^ *P* < 0.05.

**Figure 6:**
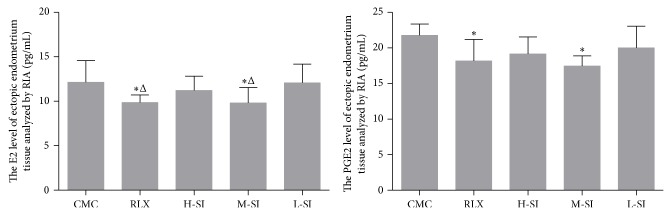
The E2 and PGE2 levels of homogenate of ectopic endometrium tissues analyzed with RIA. As is shown above, compared with CMC group (37.02 ± 5.49, *n* = 6) and L-SI group (11.42 ± 1.65, *n* = 7), the E2 levels in RLX group (9.86 ± 0.85, *n* = 6) and M-SI group (9.80 ± 1.74, *n* = 8) were significantly different. Besides, the PGE2 levels in RLX group (18.18 ± 3.00, *n* = 6) and M-SI group (17.44 ± 1.44, *n* = 8) were statistically different from CMC group (21.76 ± 1.61, *n* = 6), versus CMC group: ^*^ *P* < 0.05, versus L-SI group: ^Δ^ *P* < 0.05.

**Figure 7:**
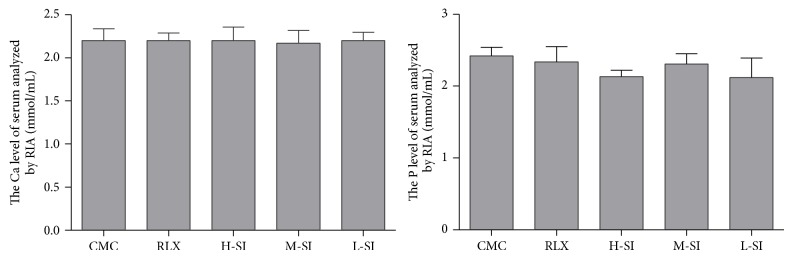
The serum levels of Ca and P analyzed with RIA. As is shown above, there were no statistical differences among groups.

According to the analysis of RIA, both of the serum and homogenate levels of E2 and PGE2 in RLX and M-SI groups were significantly different from CMC group. Besides, the differences of the serum levels of calcium (Ca) and phosphorus (P) among the five groups were not statistically significant (Figures 5–7).

### 3.5. Pathological Morphology

Light microscopic analysis showed the differences of pathological morphology between eutopic endometrium and ectopic endometrium in five-group rats after drug intervention.

**Figure 8:**
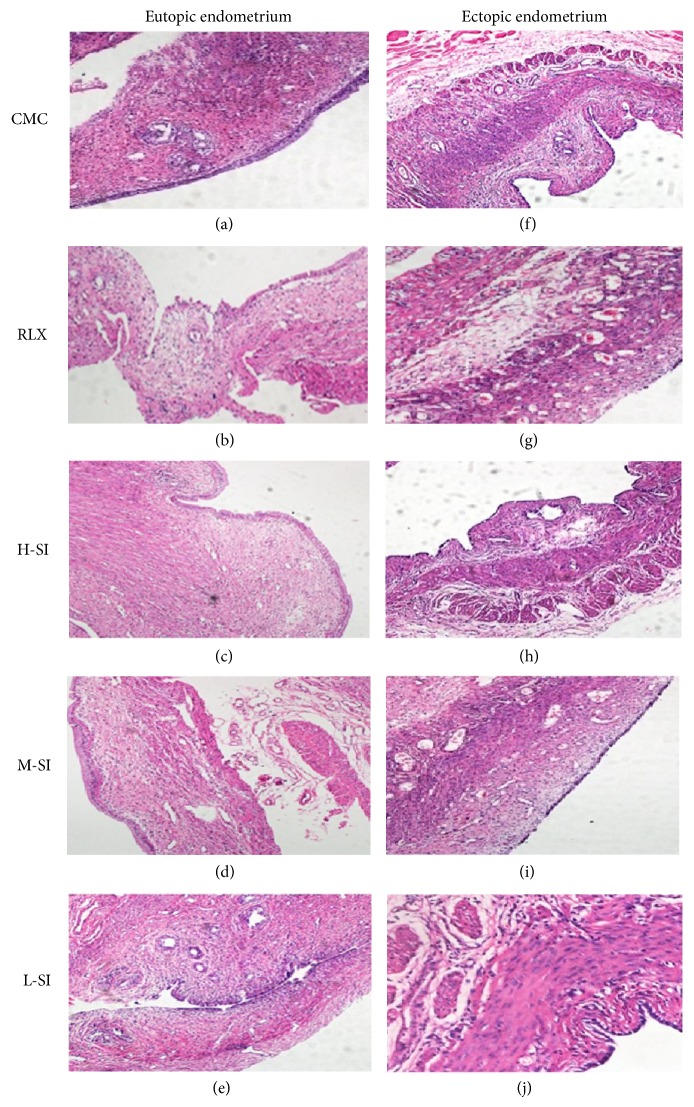
Pathological morphology of endometrium and ectopic endometrium tissues of five-group rats after drug intervention analyzed with light microscopic analysis. As is shown above, in M-SI group, the number of gland and stromal cells of ectopic endometrium was decreased and the shape of the endometrium was nearly natural which were all meaningful differences compared with the other groups.

The shape of the eutopic endometrium epithelial cells was columnar, the number of glands was natural, and the glandular epithelial cells and stromal cells were normal in both CMC group and L-SI group (Figures 8(a) and 8(e)). In RLX group and H-SI group, the shape of eutopic endometrium was irregular; the diameter of gland was shorter than that of the CMC group, while the number of glands was not obviously difference (Figures 8(b) and 8(c)). In M-SI group the columnar shape was nearly natural and the number of gland and glandular epithelial cells was almost normal (Figure 8(d)). Importantly, the epithelial cells of ectopic endometrium in CMC group and L-SI group were columnar and the number of gland and stromal cells was increased, but the diameter of gland was shortened (Figures 8(f) and 8(j)); in RLX groups, the shape of the epithelial cells of ectopic endometrium was flat, the number of gland and stromal cells decreased, and a lot of cavitations appeared (Figure 8(g)), while the shape of epithelial cells of ectopic endometrium in both L-SI group and M-SI group was low single-layer columnar and the number of gland and stromal cells decreased sharply (Figures 8(h) and 8(i)).

### 3.6. Proteins and Genes Expression

**Figure 9:**
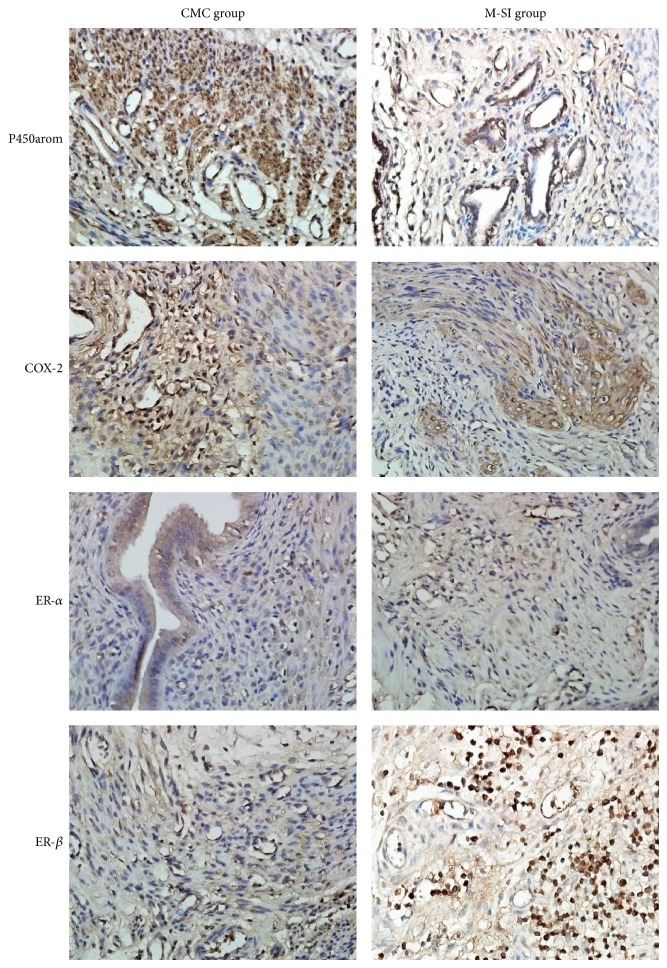
Protein P450arom, COX-2, ER-*α*, and ER-*β* analyzed with IHC. As is shown above, except for ER-*α*, the levels of P450arom and COX-2 were significantly higher in M-SI group and the level of ER-*β* was lower than that of CMC group. M-SI group (*n* = 6) versus CMC (*n* = 6): ^*^ *P* < 0.05.

**Figure 10:**
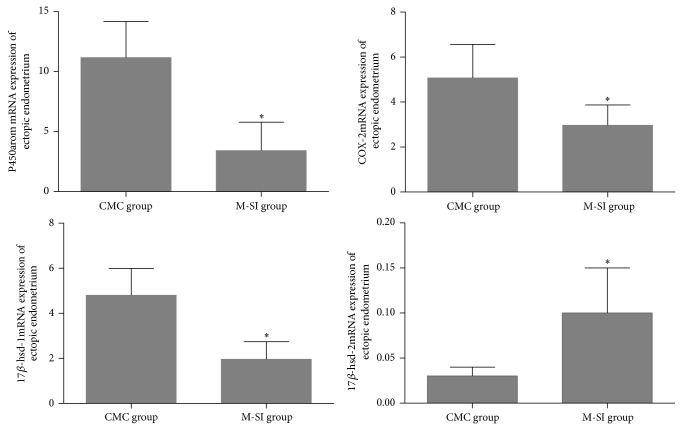
Genes expression of ectopic endometrium tissues analyzed with Real-time PCR. As is shown above, there were obviously differences of P450arom mRNA (CMC group: 11.17 ± 3.01; M-SI group: 3.42 ± 2.36), COX-2 m RNA (CMC group: 5.08 ± 1.49; M-SI group: 2.97 ± 0.90), 17*β*-hsd-1m RNA (CMC group: 4.80 ± 1.20; M-SI group: 1.96 ± 0.78), and 17*β*-hsd-2m RNA (CMC group: 0.03 ± 0.01; M-SI group: 0.10 ± 0.05). M-SI group (*n* = 6) versus CMC group (*n* = 6): ^*^ *P* < 0.05.

Comprehensively considering the effects of puerarin to treat EMT model rats, the appropriate dosage of puerarin was determined and the ectopic endometrium tissues of M-SI group were selected for the researches of mechanisms. Therefore, the genes and proteins expression of key enzymes related to estrogen secretion of ectopic endometrium tissues were detected by Real-time PCR and IHC. As a result, after puerarin intervention, the levels of protein P450arom and COX-2 were significantly decreased compared with CMC group and the level of ER-*β* was increased obviously, while the level of ER-*α* was not changed according to the statistics (Table 4 and Figure 9). In addition, except for the expression of gene 17*β*-hsd-2 mRNA which was increased, the other genes including P450arom, COX-2, and 17*β*-hsd-1mRNA were statistically decreased (Figure 10).

## 4. Discussion

Jacobson, for the first time, established the rabbit model of EMT in 1992. Rabbit, a kind of stimulating ovulation animal lacking luteal phase, is prone to infection, so the EMT rabbit model was rarely reported. Macaques and baboons are the most ideal animal model for EMT but they are limited due to their species list and low incidence of spontaneous. Besides, immunodeficiency mice can avoid the rejection reaction between dissimilar species such as being transplanted with heterogeneous (human) endometrial tissues and still keep the original morphological and biochemical characteristics; however, immunodeficiency mice cannot be used to study the changes in the body's immune just for its defect of immune function. Indeed, rats are a kind of year-round heated animal and the female ones are sensitive to sex hormone; importantly, rats have regular sexual cycle which is beneficial for the survival of transplanted endometrium and adapting to the extremely cyclical changes. Therefore, rats either SD or Wistar are suitable for EMT modeling.

In addition, EMT rat model can be divided into spontaneous model and induced model. Spontaneous EMT model is ideal for it is closest to the onset of human; however, they are too precious to get such as Rhesus monkeys and South American monkeys. Induced EMT model contains allograft and autograft. Allografts transplant the endometrial tissue into immunodeficiency animals such as nude mouse and are suitable for the etiology and pathogenesis researches of EMT, while autograft gathers the animal's own endometrium and transplants it to itself and is suitable for curative effects of drugs and the mechanisms researches of EMT.

According to our aims of experiment and the existing experimental conditions, our experiment adopted the female SD rats for EMT model with the method of autograft and the success rate was 56.06% which was lower than 75%–85% reported by reference [15]. We speculated that it might because we autologous transplanted 4 pieces endometrial tissues into 4 different locations including ovary and the root of mesenteric where were susceptible to infection in our experiment while the reference reported that the transplanted tissues only 1 or 2 pieces and the locations were common such as ligament of uterine, abdominal wall or other sites where were insusceptible to intestinal symptoms.

Puerarin, a kind of phytoestrogen, plays both estrogenic activity and anti-estrogenic activity [16]. On one hand, certain doses of phytoestrogen can produce efficiency similar to 17*β*-estradiol in body as a molecule of estrogen. Phytoestrogen, on the other hand, can preempt the receptor site and weaken the estrogen response of target cells as a molecule of antiestrogen. The ultimate effect mainly depends on the dose of the phytoestrogen, the level of endogenous estrogen in body, and the types of estrogen receptor; for example, the body is in a state of relatively high level of estrogen when women are in the reproductive period and the phytoestrogen has a role of antiestrogen activity. Our previous experiments suggest that the appropriate dose of puerarin could inhibit the growth of ectopic endometrium and has almost no side effects such as osteoporosis [17].

Puerarin could reduce the autocrine of E2 of ectopic endometrium. Researchers reported that E2 played a promoting role in the process of EMT [18, 19]. It is commonly believed that E2 could specially combine with ER of endometrial cells and the conformational changes could further promote E2-ER compounds to combine with transcription regulatory proteins. AFI and AF2 in the transcription area of ER mediate EREs on the target gene promoter and start the gene transcription, thus promoting the proliferation of ectopic endometrial cells. Therefore, the formation of high estrogen level is an important factor in promoting the growth of ectopic endometrium hyperplasia. This study has found that the levels of E2 not only in serum but also in ectopic endometrium tissues were decreased by puerarin and the growth of ectopic endometrium was suppressed.

Puerarin can inhibit the expression of P450arom and COX-2 of ectopic endometrium, reduce the levels of E2 and PGE2, and block the positive feedback mechanism of E2 synthesis. P450arom is one of the key enzymes in the progress of estrogen synthesis and it is regulated by the specific promoter. In ectopic endometrium, the highly expressed upstream stimulatory factor (USF 2) can activate steroidogenic factor-1 (SF-1); the latter combines with cAMP and can activate the genes expression signal system and promote the expression of P450arom [20]. Our study suggests that puerarin could reduce the autocrine of E2 by blocking the expression of P450arom and inhibiting the growth of ectopic endometrium. Moreover, PGE2 produced by stromal cells of ectopic endometrium could induce the expression of P450arom and stimulate its activity; simultaneously, estrogen promotes the expression of COX-2 [21], one of the key enzymes of the synthesis of PGE2. Thus, partial ectopic lesions can form a positive feedback loop, synthesize estrogen, and stimulate the growing of ectopic endometrium [22]. After puerarin intervention, both of the expression of COX-2 and the level of PGE2 were decreased, which suggests that puerarin could infect the expression of P450arom by reducing the secretion of PGE2.

Puerarin could promote the metabolism of E2 by upregulating the expression of 17*β*-hsd-2 and downregulating the expression of 17*β*-hsd-1. Dassen et al. [23] reported that 17*β* hydroxy steroid dehydrogenase 1,2,3,5,7,12 had close relationship with the hormone metabolism, especially 17*β*-hsd-1 and 17*β*-hsd-2. In human body, estrogen is mainly composed of three forms including estrone (E1), E2, and their metabolites estriol (E3). Interestingly, 17*β*-hsd-2 could transform E2 into E1 and 17*β*-hsd-1 could reverse the transformation. The results of this study show that 20 mg/kg puerarin could reduce the level of E2 by regulating the expression of 17*β*-hsd-1 and 17*β*-hsd-2.

Furthermore, it is widely regarded that the pathology of EMT is an inflammatory process. Researchers have found that ER*β* could reduce the inflammation and induce the apoptosis of ectopic endometrium tissues by rivaling the role of ER*α* [24]. Our study indicates that puerarin could upregulate the expression of ER*β* and inhibit the inflammatory process.

## 5. Conclusions

Puerarin, at a dose of 20 mg/body weight (kg)/day, has a therapeutic effect on EMT model rats and could be a potential therapeutic agent for the treatment of EMT in clinic.

## Figures and Tables

**Table 1:** The primer sequences of target genes.

Target gene	Primer sequences
GAPDH:	F5′-GTG AAG GTC GGA GTC AAC GG-3′
	R5′-CCA TCA CGC CAC AGT TTC CC-3′

P450arom:	F5′-CAG AGT ATC CGG AGG TGG AA-3′
	R5′-ACT CGA GCC TGT GCA TTC TT-3′

COX-2:	F5′-AAA GCC TCG TCA GAT GCT A-3′
	R5′-ATG GTG GCT GTC TTG GTA GG-3′

17*β*-HSD-1:	F5′-GTT ATG AGC AAG CCC TGA GC-3′
	R5′-TCT GGA TCC CCT GAA ACT TG-3

17*β*-HSD-2:	F5′-GCC CTG GTG CTC TAG AAC TG-3′
	R5′-AGT TCC ACA TCG GCC ACT AC-3′

**Table 2:** Antibodies used in this research.

Antibody	Host species	Source
P450arom	Rabbit	Santa Cruz
COX-2	Goat	Santa Cruz
ER-*α*	Rabbit	Santa Cruz
ER-*β*	Rabbit	Santa Cruz
Goat anti-rabbit IgG, rabbit anti-goat IgG		Santa Cruz

**Table 3:** The success rate of EMT rat model induced by autotransplantation.

Operation	The excluded reason		Success rate
Autotransplantation (132)	Anesthetization Infection	9 24	75.00%

Exploratory laparotomy (99)	Anesthetization Ectopic foci fester Ectopic foci are too small Without ectopic tissue growth A large amount of ascites Severe adhesion Underweight	1 12 5 2 2 2 1	74.74%

Total success rate (74/132)			56.06%

**Table 4:** The proteins levels of ectopic endometrium analyzed with IHC.

Protein	Group	Number	Positive ratio (*X*)	Optical density (*Y*)	Positive index (*X*∗*Y*)
P450arom	CMC group	6	75.77%	16.83	727.19 ± 339.58
	M-SI group	6	42.33%	9.52	353.91 ± 125.62^*^

COX-2	CMC group	6	58.83%	17.44	1008.91 ± 356.48
	M-SI group	6	46.73%	8.94	417.85 ± 143.76^*^

ER-*α*	CMC group	6	36.30%	21.73	836.93 ± 468.72
	M-SI group	6	37.49%	17.21	679.71 ± 399.73

ER-*β*	CMC group	6	51.62%	21.93	1071.25 ± 523.73
	M-SI group	6	76.28%	59.36	4646.54 ± 1777.93^*^

M-SI group versus CMC group: ^*^ *P* < 0.05.
